# The Importance of Electrostatics and Polarization for Noncovalent Interactions: Ionic Hydrogen Bonds *vs* Ionic Halogen Bonds

**DOI:** 10.1007/s00894-022-05189-6

**Published:** 2022-08-25

**Authors:** Tore Brinck, André Nyberg Borrfors

**Affiliations:** Applied Physical Chemistry, Department of Chemistry, CBH, KTH Royal Institute of Technology, 100 44 Stockholm, Sweden

**Keywords:** Hydrogen bond, Halogen bond, Electrostatic potential, Charge penetration, Intermolecular interaction

## Abstract

A series of 26 hydrogen-bonded complexes between Br^−^ and halogen, oxygen and sulfur hydrogen-bond (HB) donors is investigated at the M06-2X/6–311 + G(2df,2p) level of theory. Analysis using a model in which Br^−^ is replaced by a point charge shows that the interaction energy (${\Delta E}_{Int}$) of the complexes is accurately reproduced by the scaled interaction energy with the point charge (${\Delta E}_{Int}^{PC}$).This is demonstrated by ${\Delta E}_{Int}=0.86{\Delta E}_{Int}^{PC}$ with a correlation coefficient, *R*^*2*^ =0.999. The only outlier is (Br-H-Br)^−^, which generally is classified as a strong charge-transfer complex with covalent character rather than a HB complex. ${\Delta E}_{Int}^{PC}$ can be divided rigorously into an electrostatic contribution (${\Delta E}_{ES}^{PC}$) and a polarization contribution (${\Delta E}_{Pol}^{PC}$).Within the set of HB complexes investigated, the former varies between -7.2 and -32.7 kcal mol^−1^, whereas the latter varies between -1.6 and -11.5 kcal mol^−1^. Compared to our previous study of halogen-bonded (XB) complexes between Br^−^ and C–Br XB donors, the electrostatic contribution is generally stronger and the polarization contribution is generally weaker in the HB complexes. However, for both types of bonding, the variation in interaction strength can be reproduced accurately without invoking a charge-transfer term. For the Br^−^···HF complex, the importance of charge penetration on the variation of the interaction energy with intermolecular distance is investigated. It is shown that the repulsive character of ${\Delta E}_{Int}$ at short distances in this complex to a large extent can be attributed to charge penetration.

## Introduction

Noncovalent interactions bind molecules together in condensed phases and play important roles in chemical and biological processes. Rational design of catalysts, pharmaceuticals and supramolecular systems relies heavily on the fine-tuning of noncovalent interactions. Among the noncovalent interactions, the hydrogen-bond forms a special category due to its high strength and directional character, characteristics that are the reasons behind its frequent utilization in natural biological systems as well as in artificially designed systems. In recent years, the undisputed reign of hydrogen-bonding in rational design has become challenged by another form of noncovalent interactions. Halogen bonding shares many of the characteristics of hydrogen bonding, but it is even more highly directional in character and provides a complementary interaction to use in areas, such as drug design and supramolecular design [1, 2]. The natures of hydrogen and halogen bonding have been the focus of numerous studies, but the scientific community is still far from reaching consensus and particularly the importance of charge-transfer and covalent contributions remains under debate [3–17].

A large number of methods have been developed for analyzing intermolecular interactions in terms of well-defined and physically significant energy components [18]. This type of analysis is referred to as energy decomposition analysis (EDA). Most EDA methods are based on the supermolecular approach, i.e., variational quantum chemical calculations are performed on both the molecular complex and its isolated fragments, and the interaction energy is decomposed by the use of intermediate wavefunctions [19–27]. There are also methods in which the interaction between the fragments is treated as a perturbation to the non-interacting system [5, 22, 28, 29]. The former type of methods originates from the work of Kitaura and Morokuma in the mid-1970s [19], whereas the latter type is dominated by variants of symmetry-adapted perturbation theory (SAPT) with the first practical formulation appearing in the late 1970s [22]. Independent of the EDA type, the interaction energy is typically considered to be dominated by five energy components, i.e., exchange repulsion, electrostatics, polarization, dispersion and charge transfer. However, there are methods, such as those based on the quantum theory of atoms in molecules (QTAIM), that decompose the interaction energy into other contributions [30, 31].

Exchange or Pauli repulsion is a short-range repulsive term that stems from the overlap of the electron densities of the interacting fragments. In EDA methods based on wavefunction theory, this term is a result of the requirement of the supermolecular wavefunction to be antisymmetric with respect to exchange of electrons between the fragments, and an antisymmetry operator is introduced when constructing the first-order wavefunction from the non-interacting fragment wavefunctions. Exchange repulsion is considered a first-order term, but in SAPT it also appears at higher orders, where it is coupled to other interaction terms, such as polarization and dispersion [5, 28].

The electrostatic interaction energy is another first-order term, and it corresponds to the classical Coulomb interaction between the (static) charge distributions of the non-interacting fragments in the geometry of the molecular complex. In some EDA methods, exchange and electrostatics are not separated but considered as a single term [23, 25].

Polarization, or induction, is the lowering of the Coulombic interaction energy due to the polarization of each fragment by the charge distribution of the other. In orbital-based methods, such as Hartree–Fock or Kohn–Sham DFT, polarization results from the excitation of electrons from occupied to virtual orbitals within each fragment.

Dispersion, or London interaction, is often described as a Coulombic interaction arising from the instantaneous and mutual polarization of the charge distributions of the interacting molecules, e.g., induced dipole–induced dipole interactions. In wavefunction theory, dispersion appears as an electron correlation effect due to the contributions of configuration functions with concurrent excitations within the fragments. However, as demonstrated by Feynman, the dispersive force can be calculated from the electron density of the molecular complex and is the result of a polarization of the density of each interacting fragment [32].

Charge transfer is considered to originate from the transfer of electrons from occupied orbitals of one fragment to virtual orbitals of the other. Often it is referred to as a covalent contribution to the interaction, although as we will discuss later in this article, charge transfer and covalency are not necessarily equivalent. In many EDA methods, the charge-transfer term is not computed explicitly but obtained by subtracting out the other energy contributions from the intermolecular interaction energy. A problem with the differentiation between polarization and charge transfer is that it requires the use of an atom-centered basis set, and depends on the size and functional form of the basis set. In the limit of an infinite basis set, the charge-transfer term vanishes as the full density deformation is contained within the polarization term. Similarly, if the basis set is too small or the method is not able to account for polarization fully due to other reasons, charge transfer can be overestimated. Some EDA methods refrain from separating these two effects and combine them into a single term [5, 20, 21, 26].

In this context, it should be emphasized that the charge-transfer term of most EDA methods is different from the classical charge-transfer contribution computed by perturbation theory that is used in natural bond order (NBO) theory, as the latter is not based on the SCF-wavefunction and does not subtract out the electrostatic contribution from the charge-transfer term [33]. This typically results in a much larger magnitude of the charge-transfer energy in NBO compared to EDA analysis.

Summing up the different energy components from the EDA analysis gives the total interaction energy within the molecular complex, and it can be expressed as

1 $${\Delta E}_{Int}={\Delta E}_{Ex}+{\Delta E}_{Es}+{\Delta E}_{Pol}+{\Delta E}_{Disp}+{\Delta E}_{CT}$$

However, it should be noted that the different energy terms are generally computed from the geometry of the fragments in the molecular complex. Thus, they do not contain the energy required for deforming the fragments from their geometries in the isolated state. After adding this nuclear deformation energy term (${\Delta E}_{Nuc}$) to ${\Delta E}_{Int}$, we obtain the energy for forming the complex from the separated fragments, which we will refer to as the complexation energy (${\Delta E}_{Cmpl}$).

2 $${\Delta E}_{Cmpl}={\Delta E}_{Int}+{\Delta E}_{Nuc}$$

${\Delta E}_{Nuc}$ is typically small for weak noncovalent interactions, but generally increases with the strength of the interaction and is often large in interactions that are viewed as having covalent character.

In a recent study, we used a point-charge approach to investigate the importance of electrostatics and polarization for halogen-bond interactions between Br^−^ and halogen-bond donors of the types RC $\equiv$ CBr and R_3_CBr [15]. Although less elaborate, this point charge (PC) model has some advantages compared to the typical schemes used for EDA. The PC model gives an accurate description of electrostatics and polarization, and the two terms are rigorously defined and separated. In addition, the method is completely free of charge transfer, as the model has no electrons to transfer from the electron donor to the electron acceptor. Our study found that the ${\Delta E}_{Cmpl}$ is accurately reproduced by the PC interaction energy (${\Delta E}_{Int}^{PC}$), when the latter is scaled by a factor of 0.9 [15]. Interestingly, this shows that the variation in the ${\Delta E}_{Cmpl}$ over the whole data set is fully accounted for by only considering electrostatics and polarization. In the data set analyzed, ${\Delta E}_{Nuc}$ is generally small and varies between 0.1 and 1.3 kcal mol^−1^, and thus ${\Delta E}_{Int}$ also correlates well with the scaled ${\Delta E}_{Int}^{PC}$. Furthermore, we found that the polarization energy contributes strongly to ${\Delta E}_{Int}^{PC}$ and varies between -2.8 and -11.5 kcal mol^−1^. In some of the weakest complexes, the electrostatic interaction energy is positive and the interaction is driven by polarization.

In this study, we have used the PC model to analyze the interactions in a set of HB complexes between Br^−^ and halogen, oxygen and sulfur HB donors. We find that ${\Delta E}_{Int}$ is accurately reproduced by the scaled ${\Delta E}_{Int}^{PC}$ with a similar scaling factor as for the XB complexes studied previously. The interactions within the complexes have been analyzed and compared with XB-bonded complexes with the objective of understanding the differences and similarities between HB and XB interactions.

## Theoretical background of the PC model


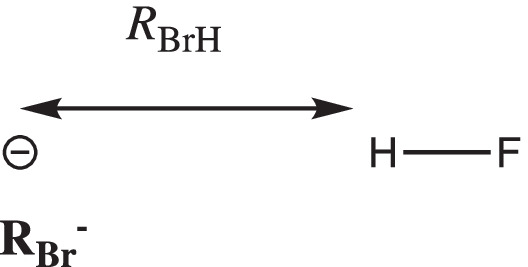
The scheme above shows the point charge (PC) model for the interaction of Br^−^ with a hydrogen-bond donor of the type XH or RXH, exemplified by the Br^−^•••HF complex. The geometry of (R)XH is obtained from the quantum chemical structure optimization of the full complex, and the point charge (*q*_Br_^−^) is placed at the position of the Br^−^ nucleus (**R**_Br_^−^) in that complex. The total interaction energy (Δ*E*^PC^) between the point charge and (R)XH is obtained as the difference in the Born–Oppenheimer energy with and without the point charge included in the Hamiltonian.

3 $${\Delta E}_{Int}^{PC}=E\left[{\widehat{H}}_{q}\left({{\varvec{R}}}_{\mathrm{Cmpl}}\right)\right]-E\left[{\widehat{H}}_{0}\left({{\varvec{R}}}_{\mathrm{Cmpl}}\right)\right]$$

The ${\Delta E}_{Int}^{PC}$ can be separated into an electrostatic term and a polarization term:

4 $$\mathrm{}{\Delta E}_{Int}^{PC}={\Delta E}_{ES}^{PC}+{\Delta E}_{Pol}^{PC}$$

The electrostatic interaction energy (${\Delta E}_{ES}^{PC}$) is computed from the electrostatic potential [*V*(**r**)] of RXH at the position of the Br^−^ in the complex.

5 $${\Delta E}_{ES}^{PC}={q}_{{Br}^{-}}{V}_{0}\left({{\varvec{r}}}_{q}\right)={-V}_{0}\left({\mathbf{R}}_{{\mathrm{Br}}^{-}}\right)$$

where we have added the subscript 0 to *V*(**r**) to emphasize that *V*_0_(**r**) is computed from the unperturbed (static) charge distribution of the HB donor.

*V*(**r**) is rigorously defined

6 $$V\left({\varvec{r}}\right)=\sum_{A}\frac{{Z}_{A}}{\left|{{\varvec{R}}}_{A}-{\varvec{r}}\right|}-\int \frac{\rho\left({{\varvec{r}}}^{^{\prime}}\right)d{\varvec{r}}}{\left|{{\varvec{r}}}^{^{\prime}}-{\varvec{r}}\right|}$$

where *Z*_A_ is the charge on nucleus A located at **R**_*A*_, and *ρ*(**r**) is the electron density function. *V*(**r**) is a physical observable and can be determined by experiment, but is more commonly computed using wavefunction theory or Kohn–Sham DFT. *V*(**r**) is a one-electron property and is, compared to, e.g., the electronic energy, relatively insensitive to the computational method or basis set. ${qV}_{0}\left({{\varvec{r}}}_{q}\right)$ corresponds to the interaction energy between a point charge *q* at **r**_*q*_ and the static (unperturbed) charge distribution of the molecule. It is the exact interaction energy within the limit of an infinitesimal charge, i.e., when polarization is negligible. $q{V}_{q}\left({{\varvec{r}}}_{q}\right)$ is the interaction energy between *q* and the perturbed charge distribution of the molecule, i.e., when the electron density is polarized due to *q* positioned at **r**_*q*_**.** However, $q{V}_{q}\left({{\varvec{r}}}_{q}\right)$ is not the complete interaction energy as there is an energy cost of polarizing the electron density, which in the linear response approximation equals minus one half of the gain in interaction energy due to the polarization.

Within the PC model, ${\Delta E}_{Pol}^{PC}$ is simply obtained by

7 $${\Delta E}_{Pol}^{PC}={\Delta E}_{Int}^{PC}-{\Delta E}_{ES}^{PC}$$

Assuming linear response, the polarization energy contribution can instead be computed from *V*_*0*_(**r**) and *V*_*q*_(**r**) as

8 $${\Delta E}_{LinPol}^{PC}=1/2q\left[{V}_{q}\left({{\varvec{r}}}_{q}\right)-{V}_{0}\left({{\varvec{r}}}_{q}\right)\right]$$

In our previous study on halogen-bonded complexes, we found that the polarization followed linear response closely; ${\Delta E}_{Pol}^{PC}$ and ${\Delta E}_{LinPol}^{PC}$ were nearly identical over the whole data set [15]. Furthermore, Eq. 8 shows that even a large polarization response only generates a smaller change in the polarization energy, e.g., a 100% increase in $V\left({{\varvec{r}}}_{q}\right)$ upon polarization results in a polarization energy that is only 50% of the electrostatic interaction energy $\left[\mathrm{if}\;V_q\left({\mathbf r}_q\right)=2V_0\left({\mathbf r}_q\right){\;\Rightarrow\;\Delta E}_{LinPol}=0.5E_{ES}\right]$.

**Fig. 1:**
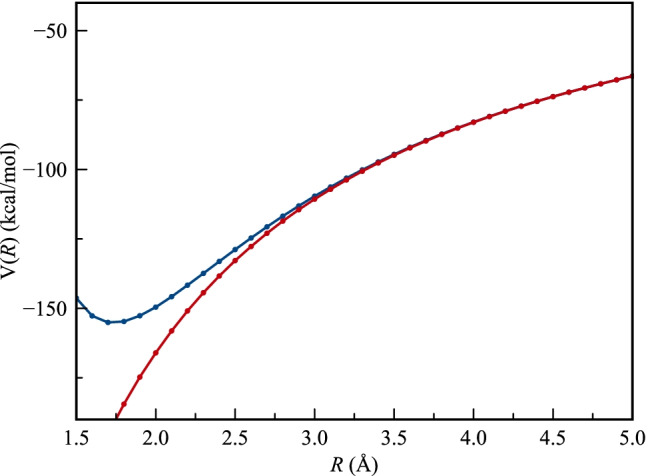
The electrostatic potential [*V*(**r**)] of Br^−^ (blue line) in kcal mol^−1^ computed at the M06-2X/6–311 + G(2df,2p) level compared to the electrostatic potential of a negative point charge of elementary charge (-e = -1 au) (red line)

Finally, we should discuss how well an anion such as Br^−^ can be represented by a negative point charge of elementary charge (-e = -1 au). Comparing their respective electrostatic potential functions, i.e., ${V}^{Br-}\left({\varvec{r}}\right)$ with ${V}^{-1}\left({\varvec{r}}\right)=-\left(1/r\right)$, we note that they both are spherically symmetric and are identical in magnitude as the radial distance from the nucleus (*r*) goes towards infinity (Fig. 1). Reducing the distance, the two functions follow each other closely but below 3 Å, ${V}^{Br-}\left(r\right)$ becomes significantly higher than ${V}^{-1}\left(r\right)$ due to an increasing amount of the electronic charge residing outside the limiting distance (*r*). At 2.5 Å, ${V}^{Br-}\left(r\right)$ is less negative than ${V}^{-1}\left(r\right)$ by 10% and around 1.7 Å, ${V}^{Br-}\left(r\right)$ reaches its minimum, where its value is 80% of ${V}^{-1}\left(r\right)$. It has been shown that this minimum coincides with the radius where exactly one electron resides outside the radius [34]. This so-called charge penetration effect has the result that Br^−^ in comparison with a negative point charge has a higher (less negative) interaction energy when interacting with a positive charge or a dipole at short distances [35, 36].

On the basis of the above analysis, it is clear that the PC model overestimates the magnitude of the electrostatic interaction energy, as well the polarization contribution to the total interaction energy due to the polarization of the electron acceptor (hydrogen or halogen bond donor). On the other hand, it does not account for the polarization of the electron donor (here Br^−^), the dispersion energy or any charge-transfer component to the interaction energy.

## Methods and procedure

The hydrogen-bonded complexes were analyzed by full structure optimization at the M06-2X/6–311 + G(2df,2p) level. The M06-2X functional is highly accurate for main-group chemistry, including noncovalent interactions, and it explicitly accounts for dispersion interaction [37]. Di Labio et al. evaluated non-counterpoise-corrected DFT interaction energies against a revision of the HB23 data set, and found the M06-2X functional to perform well with a mean absolute deviation of only 0.21 kcal mol^−1^ [38]. The 6–311 + G(2df,2p) basis set is similar in size to the basis set used by Di Labio, and is sufficiently flexible and diffuse to reduce the basis set superposition error to acceptable levels. Additional computations have been performed at the same level of theory using a point charge to represent the Br^−^ anion. These computations used the optimized geometries of the halogen-bonded complexes, but a negative point charge (-1 au) was placed at the position of Br^−^. In addition, the electrostatic potential was computed from the unperturbed density, and from the point-charge-perturbed density, of the halogen-bond donors in the geometries of the complexes. All computations were performed using the Gaussian 16 suite of software [39].

## PC model and trends in interaction energies

**Fig. 2:**
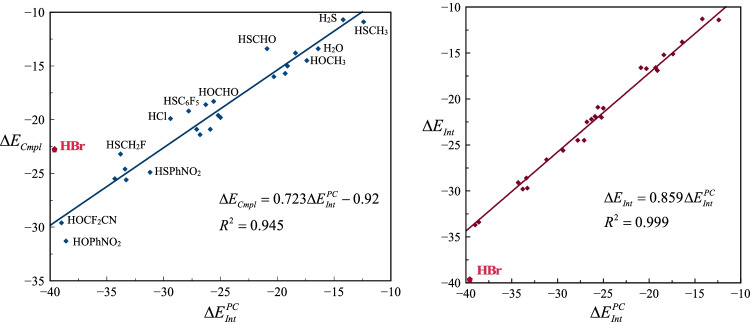
The graph to the left shows the linear correlation between ${\Delta E}_{Cmpl}$ and ${\Delta E}_{Int}^{PC}$ for the entire data set of HB complexes with Br^−^. The graph to the right shows the corresponding correlation between ∆*E*_Int_ and ${\Delta E}_{Int}^{PC}$. The complex between HBr and Br^−^ is an outlier in both correlations

The different energy components of the PC model as well as the KS-DFT computations for the entire set HB-bonded complexes are listed in Table 1. Because of the anionic HB acceptor (Br^−^), these are strong complexes and ${\Delta E}_{Cmpl}$ varies between -10.7 kcal mol^−1^ and -31.3 kcal mol^−1^. Figure 2 shows that there is a good overall linear correlation between ${\Delta E}_{Cmpl}$ and ${\Delta E}_{Int}^{PC}$ with a correlation coefficient (*R*^*2*^) of 0.94 when the (Br-H-Br)^−^ complex is excluded from the correlation. The strength of the (Br-H-Br)^−^ complex is lower than expected from the relationship. The correlation improves very significantly if the nuclear deformation energy (${\Delta E}_{Nuc}$) is subtracted out of ${\Delta E}_{Cmpl}$ to obtain ${\Delta E}_{Int}$. ${\Delta E}_{Int}$ is well reproduced by scaling ${\Delta E}_{Int}^{PC}$ with 0.86 and the correlation coefficient is excellent (*R*^*2*^ =0.999). Again (Br-H-Br)^−^ does not follow the general relation relationship, but in contrast to ${\Delta E}_{Cmpl}$, ${\Delta E}_{Int}$ is lower than predicted by the general relationship. We will return to (Br-H-Br)^−^ later but for now we will leave it out of the discussion. We note in passing that a very similar relationship was obtained in our previous study of halogen-bonded complexes with ${\Delta E}_{Int}=0.92 {\Delta E}_{Int}^{PC}$, but in that study ${\Delta E}_{Nuc}$ was low (< 1.1 kcal mol^−1^) for all complexes and there was an excellent correlation with ${\Delta E}_{Cmpl}$, as well. It is not surprising that ${\Delta E}_{Int}^{PC}$ correlates better with ${\Delta E}_{Int}$ than with ${\Delta E}_{Cmpl}$ considering that ${\Delta E}_{Nuc}$ is not included in ${\Delta E}_{Int}^{PC}$. It should also be emphasized that it is common practice to focus on ${\Delta E}_{Int}$ rather than ${\Delta E}_{Cmpl}$ when analyzing noncovalent interactions, and especially in EDA studies. However, we note that ${\Delta E}_{Nuc}$ for many of the HB complexes is relatively large and over the whole data series ${\Delta E}_{Nuc}$ varies between 0.5 and 6.6 kcal mol^−1^. There are some obvious trends in ${\Delta E}_{Nuc}$. First, ${\Delta E}_{Nuc}$ is generally larger for sulfur compared to oxygen HB donors, and increases when going from the lighter to heavier halogen atoms for halogen HB donors. Secondly, for obvious reasons, ${\Delta E}_{Nuc}$ has a tendency to increase with interaction strength. We will return to ${\Delta E}_{Nuc}$ and discuss it significance later in this article.

**Table 1:** Equilibrium Br^−^-H distance (in Å) and different energy components (in kcal mol^−1^) of the point charge interaction energy (${\Delta E}_{Int}^{PC}$) and the quantum chemical complexation energy (${\Delta E}_{Cmpl}$) for the ionic hydrogen bond complexes with Br^−^ as hydrogen-bond acceptor

	*R*_Br-H_	${\Delta E}_{ES}^{PC}$	${\Delta E}_{Pol}^{PC}$	${\Delta E}_{Int}^{PC}$	${\Delta E}_{Int}$	${\Delta E}_{Nuc}$	${\Delta E}_{Cmpl}$
HF	2.129	-23.2	-2.8	-25.9	-21.9	1.0	-20.9
HCl	1.928	-21.6	-7.8	-29.4	-25.6	5.7	-19.9
HBr	1.716	-25.3	-14.2	-39.6	-39.7	16.9	-22.7
H_2_O	2.383	-13.8	-2.6	-16.4	-13.8	0.5	-13.4
HOCH_3_	2.331	-13.5	-3.9	-17.4	-15.1	0.5	-14.5
HOCH_2_NH_2_	2.313	-14.7	-4.6	-19.3	-16.6	0.8	-15.7
HOPhNH_2_	2.232	-17.3	-7.7	-25.0	-21.0	1.2	-19.8
HOCH_2_F	2.233	-20.8	-4.4	-25.2	-22.0	2.3	-19.6
HOCHO	2.117	-20.6	-5.0	-25.6	-20.9	2.7	-18.3
HOPh	2.207	-19.1	-7.6	-26.8	-22.5	1.1	-21.4
HOCH_2_NO_2_	2.192	-27.9	-5.4	-33.3	-29.7	4.1	-25.6
HOC_6_F_5_	2.013	-25.8	-7.6	-33.4	-28.6	4.0	-24.6
HOCF_3_	2.035	-29.2	-5.1	-34.3	-29.1	3.6	-25.5
HOPhNO_2_	2.110	-29.8	-8.9	-38.6	-33.4	2.1	-31.3
HOCF_2_CN	1.989	-32.7	-6.3	-39.0	-33.7	4.2	-29.6
HSCH_3_	2.494	-7.2	-5.2	-12.4	-11.4	0.5	-10.9
H_2_S	2.286	-8.8	-5.4	-14.2	-11.3	0.6	-10.7
HSPhNH_2_	2.319	-9.2	-9.2	-18.4	-15.2	1.4	-13.8
HSCH_2_F	2.352	-12.9	-6.2	-19.1	-16.9	1.9	-15.0
HSPh	2.274	-10.9	-9.3	-20.3	-16.7	0.7	-16.0
HSCHO	2.092	-12.9	-8.1	-20.9	-16.6	3.2	-13.4
HSCF_3_	2.030	-17.7	-8.5	-26.3	-22.2	3.6	-18.6
HSCH_2_NO_2_	2.322	-19.7	-7.3	-27.1	-24.5	3.6	-20.9
HSC_6_F_5_	2.013	-16.3	-11.5	-27.8	-24.5	5.3	-19.2
HSPhNO_2_	2.137	-20.1	-11.1	-31.2	-26.6	1.7	-24.9
HSCF_2_CN	1.906	-22.5	-11.3	-33.8	-29.8	6.6	-23.2

The excellent agreement between ${\Delta E}_{Int}$ and the scaled ${\Delta E}_{Int}^{PC}$ is remarkable considering that the data set consists of a diverse group of halogen, oxygen and sulfur HB, donors and considering the simplicity of the PC model, which only accounts for electrostatics and the polarization of the HB donor. There is only a fair correlation between the electrostatic interaction energy (${\Delta E}_{ES}^{PC}$) and ${\Delta E}_{Int}$ (*R*^*2*^ =0.89), but much better correlations with ${\Delta E}_{ES}^{PC}$ are obtained if groups of HB donors of similar type, e.g., alcohols, are considered separately. This shows that caution should be used when interpreting the character of intermolecular interactions based on the analysis of a group of congeneric molecules. The polarization energy is always smaller in magnitude than the electrostatic energy, but ${\Delta E}_{Pol}^{PC}$ varies considerably, both in value (− 2.8 to − 11.5 kcal mol^−1^) and in terms of its relative contribution (11- 42%) to ${\Delta E}_{Int}^{PC}$. It should be noted that the large importance of polarization is a consequence of the anionic HB acceptor; in complexes with neutral HB acceptors, we see much smaller relative contributions from polarization and the interactions are clearly dominated by electrostatics [40]. There are similar but more distinct trends in the variation of ${\Delta E}_{Pol}^{PC}$ compared to the variation of ${\Delta E}_{Nuc}$. ${\Delta E}_{Pol}^{PC}$ is lower for the sulfur HB donors when compared to the corresponding oxygen donors, as well as for HCl compared to the oxygen donors. It also decreases with the polarizability and the conjugation of the R group. Furthermore, ${\Delta E}_{Pol}^{PC}$ generally decreases with the strength of the interaction.

## Energy decomposition along the potential-energy surface

In order to understand the relevance of the different energy components and the overall correlation between ${\Delta E}_{Int}$ and ${\Delta E}_{Int}^{PC}$, we will analyze the potential-energy surfaces of some of the complexes by studying the variation of the energy components with varying distance to the Br^−^. We will begin with the HF complex, and then continue with HCl, which has a similar ${\Delta E}_{Cmpl}$ but ${\Delta E}_{Pol}^{PC}$ and ${\Delta E}_{Nuc}$ of larger magnitudes.

**Fig. 3:**
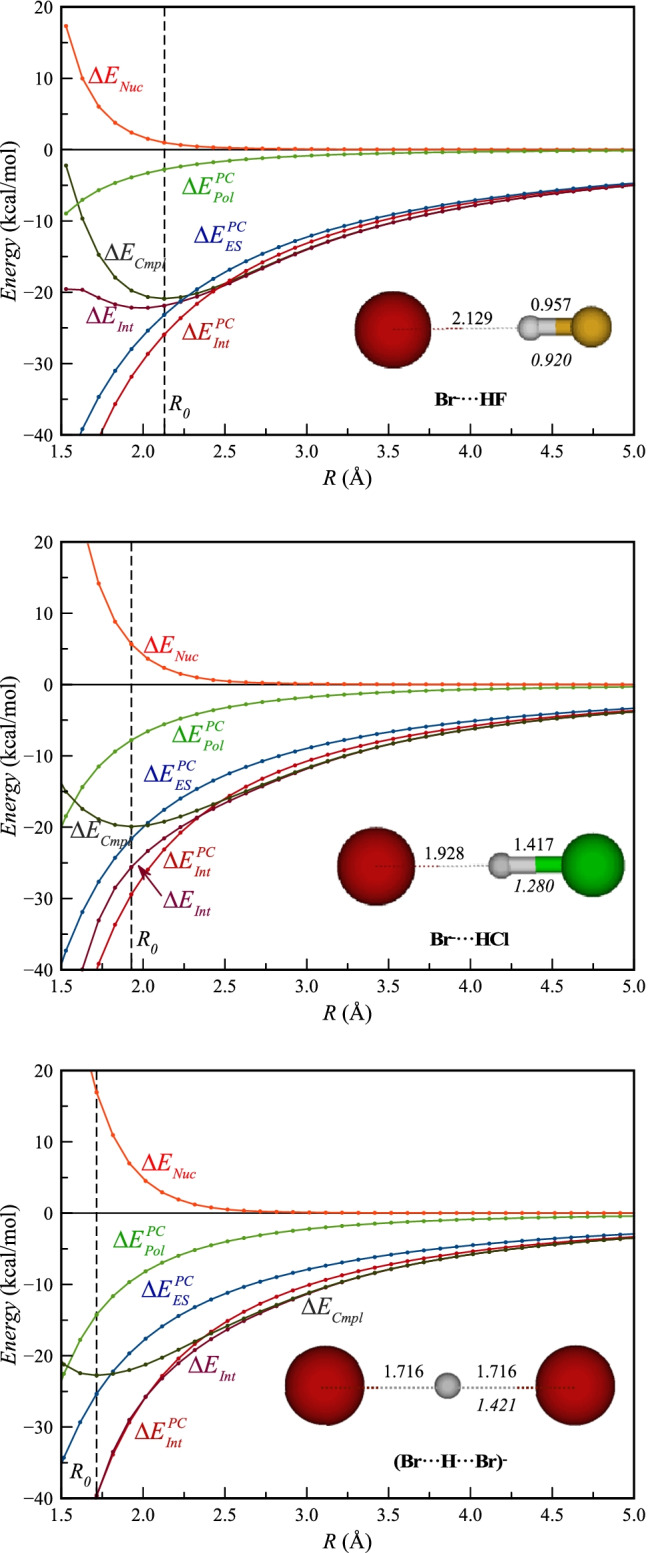
The different energy components from the full quantum chemical model and the PC model as functions of the Br-H distance (*R*) for complexes of HF, HCl and HBr with Br^−^. The vertical dotted line marks the Br···HX distance (*R*_*0*_) at the complex minimum. The corresponding structure is shown as an inset, and the X–H distance in italics is for the free hydrogen bond donor

When analyzing the HF complex in Fig. 3, we note that at *R* distances greater than 4 Å ${\Delta E}_{Cmpl}$, ${\Delta E}_{Int}$, ${\Delta E}_{Int}^{PC}$ and ${\Delta E}_{ES}^{PC}$ follow each other closely, whereas ${\Delta E}_{Pol}^{PC}$ and ${\Delta E}_{Nuc}$ are negligible. This shows that the interaction is almost entirely electrostatic at these longer distances. At distances shorter than 4 Å, ${\Delta E}_{Pol}^{PC}$ starts to decrease, leading to an increasing separation between ${\Delta E}_{Int}^{PC}$ and ${\Delta E}_{ES}^{PC}$, but ${\Delta E}_{Pol}^{PC}$ remains above -1.0 kcal mol^−1^ down to *R* = 2.8 Å. ${\Delta E}_{Cmpl}$ and ${\Delta E}_{Int}$ also become slightly lower than ${\Delta E}_{Int}^{PC}$ below 4 Å, but the difference never exceeds 1.0 kcal mol^−1^. This difference can be attributed to polarization of Br^−^ and dispersion, interactions that are not included in the PC model. ${\Delta E}_{Nuc}$ remains negligible until below 2.5 Å and reaches a value of 1.0 kcal mol^−1^ at the potential energy minimum (*R* = 2.13 Å); Consequently, ${\Delta E}_{Int}$ is 1.0 kcal mol^−1^ lower than ${\Delta E}_{Cmpl}$ at the minimum. After the minimum, ${\Delta E}_{Nuc}$ increases steadily, and this is the main reason for the increase in ${\Delta E}_{Cmpl}$ at short distances. Just before the minimum at 2.2 Å, ${\Delta E}_{Int}^{PC}$ becomes lower than ${\Delta E}_{Int}$, mainly due to increasing charge penetration, and at the minimum, ${\Delta E}_{Int}^{PC}$ (-29.4 kcal mol^−1^) is 3.8 kcal mol^−1^ lower than ${\Delta E}_{Int}$. At this point, ${\Delta E}_{ES}^{PC}$ is -23.2 kcal mol^−1^ and ${\Delta E}_{Pol}^{PC}$ is -2.8 kcal mol^−1^, clearly showing that this interaction is dominated by electrostatics and has only a minor contribution from polarization.

When analyzing the HCl complex at distances beyond 4 Å, we see similar behavior as for HF, with ${\Delta E}_{Int}$ (and ${\Delta E}_{Int}^{PC}$) completely dominated by the electrostatic term (${\Delta E}_{ES}^{PC}$). However, below 4 Å polarization starts to build up and ${\Delta E}_{Pol}^{PC}$ decreases with decreasing *R* more strongly than for HF. At the minimum (*R* = 1.93 Å), ${\Delta E}_{Pol}^{PC}$= -7.8 kcal mol^−1^ and constitutes 30% of ${\Delta E}_{Int}^{PC}$; the corresponding contribution for HF is only 10%. However, when we analyze the behavior of the sum of electrostatic and polarization terms (${\Delta E}_{Int}^{PC}$) compared to ${\Delta E}_{Int}$ it is almost identical to that in HF; below 4 Å, ${\Delta E}_{Int}$ becomes slightly more negative than ${\Delta E}_{Int}$ (maximum difference 1.0 kcal mol^−1^), and below 2.2 Å, the curves cross due to increasing charge penetration. ${\Delta E}_{Nuc}$ varies in similar manner with distance as for HF, but its magnitude is much larger at all distances, and at the complex minimum it reaches a value of 5.7 kcal mol^−1^. ${\Delta E}_{Nuc}$ alone is the reason for the minimum in ${\Delta E}_{Cmpl}$, as the gradient of ${\Delta E}_{Int}$ is everywhere positive in the range of distances investigated. In other words, the ${\Delta E}_{Int}$ curve is attractive at all distances. The large value of ${\Delta E}_{Nuc}$ is a consequence of that the H-Cl bond length increases from 1.28 Å in the free HCl molecule to 1.42 Å in the complex. Comparing the HCl complex with the HF complex, the main differences are lower ${\Delta E}_{Pol}^{PC}$ and higher ${\Delta E}_{Nuc}$ in the former.

$$\mathrm{Br}^\circleddash\;\mathrm H-\mathrm{Br}\leftrightarrow\mathrm{Br}-\mathrm H\;\mathrm{Br}^\circleddash$$

As already mentioned, (Br-H-Br)^−^ is the only complex that does not follow the scaling relationship between ${\Delta E}_{Int}$ and ${\Delta E}_{Int}^{PC}$. This is not surprising, considering that it is very far from a typical hydrogen-bonded complex, with the Lewis structure representation consisting of two equivalent resonance structures, each with a -1 charge on opposite bromines. The optimized structure with two equal bond lengths of 1.715 Å (compared to 1.421 Å in HBr) confirms the Lewis structure picture of approximately a 0.5 covalent bond order for each H-Br bond and the partial electron transfer from Br^−^ to the other Br upon complex formation. In order to better understand the potential effect of charge transfer and change in covalency, we have analyzed the potential-energy surface in a similar manner as for the HF and HCl complexes. Going from HCl to HBr follows a very similar trend as going from HF to HCl in terms of the evolution of the different energy components with decreasing *R* distance. In particular, there is a consistent increase in magnitude of ${\Delta E}_{Nuc}$ and ${\Delta E}_{Pol}^{PC}$ going from HF via HCl to HBr. However, it should be noted that for HBr, ${\Delta E}_{Nuc}$ is very high, 16.9 kcal mol^−1^, more than 2.5 times higher than for any of the other hydrogen-bonded complexes in Table 1. In addition, HBr stands out from HF and HCl in that ${\Delta E}_{Int}^{PC}$ does not become lower than ${\Delta E}_{Int}$ at distances below 2.2 Å, but instead the two energies are nearly identical at the shorter distances. This could be interpreted as an additional energy contribution from charge transfer to ${\Delta E}_{Int}$ that is building up at the shorter distances, a contribution that is missing from ${\Delta E}_{Int}^{PC}$. Alternatively, it could be viewed as that the repulsive contributions to ${\Delta E}_{Int}$ are weakened compared to HCl, following the same trend as going from HF to HCl.

We have also analyzed the potential-energy surfaces for the Br^−^ complexes with CF_3_OH and CF_3_SH. First, it can be noted that these surfaces and the variations of the different energy components with *R* are very similar in appearance as for the surfaces of HF and HCl. This clearly indicates that the character of the hydrogen bonding in oxygen and sulfur HB donors is not fundamentally different from that of the halogen HB donors. Analyzing CF_3_OH first, we find that it forms a stronger complex (${\Delta E}_{Cmpl}$= -25.5 kcal mol^−1^) than HF and HCl, and the interaction is dominated by electrostatics. At the minimum, ${\Delta E}_{Pol}^{PC}$= -7.8 kcal mol^−1^ and constitutes 15% of ${\Delta E}_{Int}^{PC}$. This can be compared to 10% and 30%, respectively in the HF and HCl complexes. ${\Delta E}_{Nuc}$ is 3.6 kcal mol^−1^, which also is intermediate between HF and HCl. Thus, it is clear that the higher strength of the CF_3_OH complex is an effect of a much stronger electrostatic interaction.

Turning to CF_3_SH, it forms a weaker complex than not only CF_3_OH but also HF and HCl. However, ${\Delta E}_{Pol}^{PC}$ (-8.5 kcal mol^−1^) is larger in magnitude than for CF_3_OH and HF, and the relative contribution, 41% of ${\Delta E}_{Int}^{PC}$, is even larger than for HCl. Thus, the weaker interaction of CF_3_SH compared to the other is the effect of a weaker electrostatic interaction. The value of ${\Delta E}_{Nuc}$ at the minimum is nearly identical to that in CF_3_OH, but it plays a larger relative role for ${\Delta E}_{Cmpl}$ in CF_3_SH compared to CF_3_OH.

## Polarization and charge transfer

The excellent agreement between the supermolecular interaction energy and the scaled point-charge interaction energy shows that the variation of the interaction energy within the data set is fully reproduced by considering only electrostatics and polarization (remember that the PC description is completely free of charge transfer, as there are no electrons that can be transferred to the HB donor). This result may seem surprising considering the character of these interactions, and particularly in some complexes, such as Br^−^···HCl, a significant charge-transfer contribution to the interaction energy could have been anticipated. There is no doubt that any EDA method that differentiates between charge transfer and polarization will indicate a charge-transfer component to the Br^−^···HCl complex, although the actual size of that component will depend upon the choice of method and basis set.

The results of the PC model could be used to argue that charge transfer plays a very minor role for the strength of these complexes. However, we rather view it as a manifestation of that these interaction terms are difficult to separate and that any division between them is arbitrary. In the PC model, the interactions must be described as electrostatics and polarization, because the model does not allow for charge transfer. On the other hand, some EDA methods will find a very significant contribution from electron transfer between the fragments as a consequence of the functional form of the atom-centered basis set. In this context, one could ask whether the failure of the PC model to describe (Br-H-Br)^−^ should be taken as evidence that a separate charge-transfer term is needed to reproduce the interaction energy of this complex. It should first be remembered that the PC model does not allow for any polarization of Br^−^. In addition, the polarization of the H-Br unit is limited by the flexibility of the basis set used in the calculation. Still, the question remains whether a model that allows for full polarization of both Br^−^ and H-Br in the geometry of the (Br-H-Br)^−^ complex would reproduce the charge distribution of the (Br-H-Br)^−^ complex as well as its interaction energy. However, such a model would require an extremely large and diffuse basis set for each fragment, which would make it impossible to distinguish polarization from charge transfer. In this context, we like to emphasize that the charge-transfer character of an interaction should not be equated with the covalent character of an individual bond. There is no doubt that the two Br-H bonds in (Br-H-Br)^−^ have an equally strong covalent character, and this results from one covalent bond being weakened and another partly covalent bond being formed upon the interaction between Br^−^ and H-Br. In the same manner, we would argue that the Br-H bond in the Br^−^···HCl complex has a partial covalent character, even though we find that the interaction energy is equally well described by the scaled PC-energy as interactions with a much smaller covalent character.

## The nuclear deformation energy as an indicator of charge transfer

**Fig. 4:**
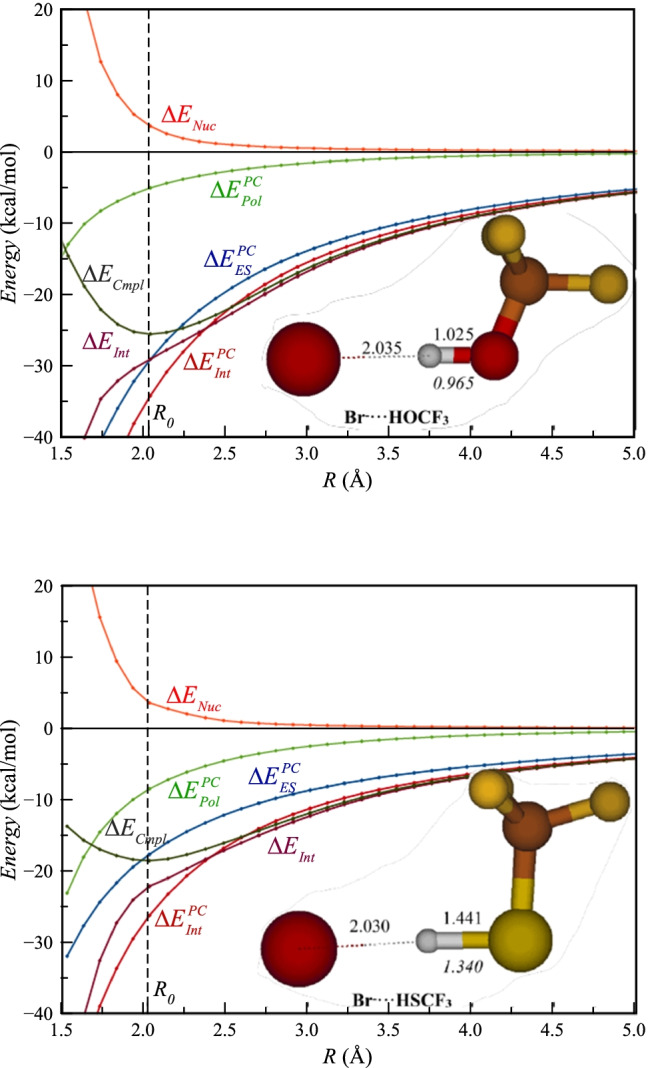
The different energy components from the full quantum chemical model and the PC model as functions of the Br-H distance (*R*) for complexes of CF_3_OH and CF_3_SH. The vertical dotted line marks the Br···H distance (*R*_*0*_) of the lowest energy structure. The lowest energy structure is shown as an inset, and the X–H distance in italics refers to the free hydrogen bond donor

**Fig. 5:**
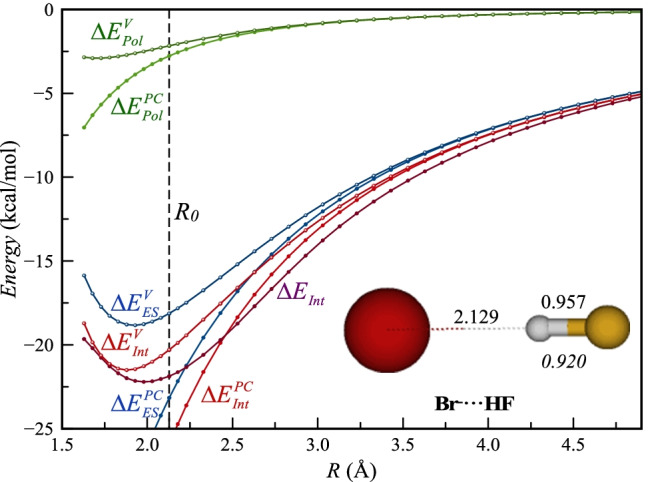
The graph shows the effect of charge penetration on the different energy components of the PC as functions of the Br···H distance (*R*) for the complexes with HF. Charge penetration corrected energies are marked with superscript *V* and compared to the PC energies and to ${\Delta E}_{Int}$. Similarly to ${\Delta E}_{Int}$, the corrected energy curves, ${\Delta E}_{ES}^{V}$ and ${\Delta E}_{Int}^{V}$, are repulsive at short distances

Another energy term that is interesting to analyze is the nuclear deformation energy ${\Delta E}_{Nuc}$. It plays a very significant role for the potential-energy surfaces presented in Figs. 4 and 5, and with the exception of the HF complex, the ${\Delta E}_{Nuc}$ contribution is solely responsible for the minimum in the ${\Delta E}_{Cmpl}$ and the repulsive character of ${\Delta E}_{Cmpl}$ at shorter distances. This is different from the typical behavior of noncovalent interactions, where ${\Delta E}_{Nuc}$ generally is of minor importance at the minimum. It should be remembered that the driving force for the nuclear deformation is to lower the total energy, and the increase in ${\Delta E}_{Nuc}$ at short distances is compensated by a larger decrease in ${\Delta E}_{Int}$. Thus, it may be more appropriate to use the term nuclear relaxation rather than nuclear deformation. The PC model takes the nuclear relaxation into account as the interaction energy is calculated using the geometry of the supramolecular complex. This is the same approach that is commonly used in supramolecular EDA methods as well as in SAPT. However, the PC model fails to reproduce the nuclear relaxation in the sense that optimizing the HB donor in the presence of the point charge will generate a much smaller deformation of the HB donor than the deformation that is induced by the presence of Br^−^. It could be argued that the nuclear relaxation of the complex results from an electron donation from Br^−^ into virtual orbitals of the HB donor, followed by a rehybridization of the occupied orbitals. Such a behavior would also imply that ${\Delta E}_{Nuc}$ could be used as an indicator of the size of the charge-transfer contribution to an intermolecular interaction. This interpretation finds partial support from the very large magnitude of ${\Delta E}_{Nuc}$ in the (Br-H-Br)^−^ complex, which clearly fulfils the criteria for a strong charge-transfer complex with a significant covalent contribution to the bonding. However, it cannot, for example, explain the large variation in ${\Delta E}_{Nuc}$ of the complexes with sulfur HB donors, as it seems unlikely that the much larger ${\Delta E}_{Nuc}$ of HSCF_2_CN (6.6 kcal mol^−1^) compared to HSPhNO_2_ (1.7 kcal mol^−1^) reflects a much larger charge-transfer contribution in the former complex. Furthermore, in our earlier study of XB bond complexes involving C–Br groups, ${\Delta E}_{Nuc}$ was generally much smaller than for the HB complexes of this study. The small ${\Delta E}_{Nuc}$ values seem to mainly reflect the character of the C − Br bond, and should not be taken to indicate a lower contribution from charge transfer in XB complexes with Br^−^ compared to HB complexes with Br^−^. In line with this conclusion, we note that XB complexes involving Br^−^ and dihalogens, such as Br_2_ or BrF, have rather large ${\Delta E}_{Nuc}$ values.

## Effect of charge penetration on the potential-energy surface

In connection to the discussion about the importance of electrostatic and polarization versus charge transfer, it should be emphasized that the PC model overestimates the magnitudes of the former contributions at shorter distances due to the neglect of charge penetration. This is the main reason that ${\Delta E}_{Int}^{PC}$ is lower than ${\Delta E}_{Int}$ at distances below around 2.2 Å, as is found for all complexes in Figs. 3 and 4. However, other energy contributions, such as exchange repulsion, could also potentially contribute to ${\Delta E}_{Int}$ and increase its value at short distances. In the case of the HF complex, ${\Delta E}_{Int}$ has repulsive character with a negative gradient at distances below 2.0 Å; a behavior that is distinctly different from ${\Delta E}_{Int}^{PC}$, which has an increasingly positive gradient with decreasing distance. Thus, it is interesting to investigate how the neglect of charge penetration affects the electrostatic and polarization contributions of ${\Delta E}_{Int}^{PC}$ along potential-energy surface of the HF complex. The HF molecule is well suited for investigating these effects, as its charge distribution can be well approximated by an atomic monopole expansion, i.e., a positive charge on H and negative charge of the same magnitude on F. On the basis of this approximation, we can estimate the charge penetration corrected energies, ${\Delta E}_{ES}^{V}$ and ${\Delta E}_{Pol}^{V}$, by

9 $${\Delta E}_{ES}^{V}={k}_{HF}^{V}{\Delta E}_{ES}^{PC}$$

and

10 $${\Delta E}_{Pol}^{V}={k}_{HF}^{V}{\Delta E}_{Pol}^{PC}$$

where

11 $${k}_{HF}^{V}=\frac{{V}^{Br-}\left({\mathbf{r}}_{\mathrm{H}}\right)-{V}^{Br-}({\mathbf{r}}_{\mathrm{F}})}{{V}^{-1}\left({\mathbf{r}}_{\mathrm{H}}\right)-{V}^{-1}({\mathbf{r}}_{\mathrm{F}})}$$

In Eq. 11, ${V}^{Br-}\left({\mathbf{r}}_{\mathrm{H}}\right)$ is the electrostatic potential of Br^−^ at the position of the H nucleus in the complex and ${V}^{Br-}\left({\mathbf{r}}_{\mathrm{H}}\right)$ is the corresponding value at the position of the F nucleus. The ${V}^{-1}\left({\mathbf{r}}_{\mathrm{H}/F}\right)$ terms refer to the electrostatic potential of a point charge (-e = -1 au) placed at the position of Br^−^. Equation 9 is exact within the approximation that the charge distribution of HF can be approximated by a monopole expansion. Equation 10 is based on the additional approximation that the exact charge distribution of Br^−^ polarizes the charge distribution of HF to the same extent as a point-charge representation. This additional approximation is likely to lead to an underestimation of ${\Delta E}_{Pol}^{V}$ and thus also the predicted ${\Delta E}_{Int}^{V}$ should be too low. However, the magnitude of the polarization component is smaller than that of the electrostatic component for the HF complex, and thus, the resulting error should have a relatively small impact on the size of ${\Delta E}_{Int}^{V}$. In Fig. 5, we compare ${\Delta E}_{ES}^{V}$, ${\Delta E}_{Pol}^{V}$ and ${\Delta E}_{Int}^{V}$ with the corresponding PC energies as well as ${\Delta E}_{Int}^{V}$ as functions of the intermolecular distance Br-H. First, we note that ${\Delta E}_{ES}^{V}$ and ${\Delta E}_{Int}^{V}$ already begin to deviate significantly from the corresponding PC values at about 3.5 Å, whereas for ${\Delta E}_{Pol}^{V}$ it is not until around 2.5 Å that the deviation becomes significant. ${\Delta E}_{Int}^{V}$ consistently lies above ${\Delta E}_{Int}$ down to a distance of about 1.8 Å, where the two curves approach each other. This energy difference can mainly be attributed to dispersion and polarization of Br^−^, energy components that are not included in ${\Delta E}_{Int}^{V}$. At the complex minimum (*R* = 2.13 Å), ${\Delta E}_{Int}$ is 1.6 kcal mol^−1^ lower than ${\Delta E}_{Int}^{V}$, which is in line with the expected energy contribution from dispersion and polarization of Br^−^. ${\Delta E}_{Int}$ also continues to decrease after the minimum in ${\Delta E}_{Cmpl}$ and reaches its minimum at 1.98 Å, after which the energy increases. The general increase in ${\Delta E}_{Int}$ at shorter distances for noncovalent interactions is attributed to exchange repulsion in most EDA methods. Therefore, it is interesting to note that ${\Delta E}_{ES}^{V}$ behaves similarly to ${\Delta E}_{Int}$ and has a minimum at a similar distance, despite that ${\Delta E}_{ES}^{V}$ lacks a contribution from exchange repulsion; the increase energy at shorter distances is instead a result of charge penetration. ${\Delta E}_{Int}^{V}$, which also lacks exchange repulsion, behaves similarly to ${\Delta E}_{Int}$ and ${\Delta E}_{ES}^{V}$, but reaches its minimum at a slightly shorter distance.

## Difference between hydrogen and halogen bonding

Finally, we discuss the difference in character of HB versus XB bonding based on the analysis with the PC model. First of all, it should be noted that we have found very similar relationships between ${\Delta E}_{Int}$ and ${\Delta E}_{Int}^{PC}$ for the two types of bonding in the data sets that we have investigated, i.e., ${\Delta E}_{Int}$ ≈ 0.9 ${\Delta E}_{Int}^{PC}$. This agreement is encouraging, as it indicates that the high correlations are not fortuitous but rather that the PC model reflects the physics of the interactions. Secondly, as already noted, we find a much larger variation in ${\Delta E}_{Nuc}$ for the HB data set, where it varies between 0.5 and 6.6 kcal mol^−1^. In the XB data set, the variation is only between 0.1 and 1.3 kcal mol^−1^, which we attribute to the rigidity of the C–Br bond. Instead, we find that the most significant difference between the HB and XB complexes is in the relative contributions of polarization and electrostatics. In the HB data set, the contribution of ${\Delta E}_{Pol}^{PC}$ to ${\Delta E}_{Int}^{PC}$ varies between − 2.8 and − 11.5 kcal mol^−1^ in absolute terms and between 11 and 42% in relative terms, whereas for the XB data set the corresponding numbers are -4.0 to -9.9 kcal mol^−1^ and 29% to 880%. The reason for the extremely large relative contributions of ${\Delta E}_{Pol}^{PC}$ in some XB complexes is that these are weakly bonded XB complexes where the electrostatic contribution is positive and thus is counteracted by a strongly negative ${\Delta E}_{Pol}^{PC}$. Thus, overall, we find that polarization has much higher importance for XB compared to HB, and this is the main difference in the character of the two types of bonding. We have found no indications that a separate charge-transfer term is needed to describe the difference between halogen and hydrogen bonding.

## Summary and conclusion

In this study, we have shown that the PC model describes the variation of the halogen bond interaction energy accurately within a diverse group of hydrogen-bond donors and their complexes with Br^−^, as indicated by the relationship ${\Delta E}_{Int}$=0.86 ${\Delta E}_{Int}^{PC}$ with *R*^*2*^ =0.999. The excellent correlation is remarkable considering that the PC model only accounts for electrostatics and polarization, and considering the large variation in chemical structure; the data set includes halogen, oxygen and sulfur HB donors, and the two latter feature both electron donating and accepting substituents. The only complex that does not follow the general correlation is (Br-H-Br)^−^. This is not surprising considering that this complex is classified as a strong charge-transfer complex with a large covalent character rather than an HB complex. However, the failure of the PC model to reproduce the interaction energy of this complex can partly be ascribed to incomplete description of polarization.

The different energy components for the PC model and the full quantum chemical model have been investigated along the potential-energy surface (PES) for the complexes of five HB donors. In all complexes, we find that the long-range interaction is dominated by electrostatics, and that ${\Delta E}_{Int}^{PC}$ approximately follows ${\Delta E}_{Int}$ down to around 2.2 Å. With the exception of the (Br-H-Br)^−^ complex, ${\Delta E}_{Int}^{PC}$ becomes lower than ${\Delta E}_{Int}$ below 2.2 Å, due to increasing charge penetration at shorter distances. In all complexes, except for the complex with HF, ${\Delta E}_{Int}$ has a positive gradient at all distances, and ${\Delta E}_{Nuc}$ defines the repulsive character of ${\Delta E}_{Cmpl}$ at short distances. An investigation of the charge-penetration effect on the electrostatic and polarization energies in the HF complex indicates that the repulsive component to ${\Delta E}_{Int}$ of this complex can to a great extent be attributed to charge penetration and that the contribution from exchange repulsion is relatively minor.

The results of the current study have been compared to our previous study on halogen bonding complexes between Br^−^ and XB bond donors of the types RC $\equiv$ CBr and R_3_CBr [15]. In that study, we found a very similar relationship between ${\Delta E}_{Int}$ and ${\Delta E}_{Int}^{PC}$, i.e., ${\Delta E}_{Int}$=0.92 ${\Delta E}_{Int}^{PC}$. The similar scaling factor of the two studies supports the conclusion that the PC model is able to to describe the physics behind the interactions. The main difference in the potential-energy surfaces of the two data sets is in the ${\Delta E}_{Nuc}$ term, and it generally is smaller and varies less in the XB bonding data set. Our interpretation is that the difference is a reflection of the character of the C–Br bond rather than a consequence of a larger charge-transfer contribution in the hydrogen-bond interactions. The main differences in the character of the bonding between the HB- and XB-bonded complexes are instead a larger influence of electrostatics and smaller contribution from polarization in the former type. However, we do not find it necessary to invoke charge transfer in order to understand the difference in character between ionic hydrogen and halogen bonding.

It may seem that the results of this and our previous study using the PC model are to some extent at odds with the conclusions typically drawn from supermolecular EDA and SAPT. However, there is a lack of studies where those type of methods have been used to analyze strong ionic hydrogen and halogen bonds of the types studied here. We hope that someone will pick up the baton, and employ such methods to help us increase the understanding of these important interactions on the border between noncovalent interactions and covalent bonding.

## Data Availability

Not applicable.

## Questions/Comments

**Szalewicz comment:**

*No need for charge transfer:* The authors show that interactions of Br− with hydrogen-bond (HB) donors can be described without invoking charge transfer interactions. This agrees with the assertion in my contribution that the concept of charge transfer is hardly needed to describe non-covalent interactions (NCIs).

**Szalewicz asks:** *What are Coulomb interactions?*: The quantum mechanical Hamiltonian for a system of nuclei treated as point charges and electrons contains only Coulomb interactions. Thus, one might say that all types of interactions, including NCIs, are just Coulomb interactions. Consequently, one could in principle say, as the authors do, that not only electrostatic, but also induction and dispersion interactions are Coulomb interactions. However, in my opinion, it is better to restrict this phrase to electrostatic interactions only, which are directly given by the Coulomb energy law defining interactions energy between static charge distributions.

**Answer:** We agree all the potential energy terms of the molecular Hamiltonian stem from Coulombs law and in that sense all interactions, including NCI, may be considered Coulombic. However, we like to use the term electrostatic interactions to separate the classical Coulomb interactions between the static (unperturbed) charge distributions of the molecules from other interaction terms, such as polarization or dispersion. In the terms of perturbation theory, electrostatic interactions are first order whereas other Coulomb interactions are of higher order. The use of electrostatic instead of Coulomb may seem like a purely semantic choice, but it is used to avoid misunderstandings.

**Szalewicz asks** *Induction energy:* In the discussion of induction interactions, the authors say on pages 3 and 6 that the induction energy is the Coulomb energy resulting from interaction involving deformed densities, but multiplied by a factor 1/2 to account for the costs of deformation. As I point out in my contribution, in the rigorous quantum approach that latter quantity does not appear and the factor 1/2 results from the fact that the induction energy is a second-order quantity in the interaction operator *V* , while the electrostatic energy is a quantity of the first order.

**Answer:** We agree. When we calculate the induction energy, or polarization energy ($\Delta E^{PC}_{Pol}$) as we call it, using Eq. 7, i.e. as the difference between the full PC interaction energy ($\Delta E^{PC}_{Int}$) and the PC electrostatic energy ($\Delta E^{PC}_{ES}$), the $\Delta E^{PC}_{Pol}$ is obtained without introducing the factor 1/2. Equation 8, which invokes the factor 1/2, gives the second-order expression for the polarization energy ($\Delta E^{PC}_{LinPol}$) . In the studied systems, Eq. 7 and Eq. 8 give almost identical values of the polarization energy, indicating that the interactions are very well described already at second order, and that higher orders of polarization are nearly negligible.

**Szalewicz asks:** The authors state in the abstract and on page 2 that the repulsive character of interaction energy at short distances can be attributed to charge penetration. While charge penetration is often (but not always) reducing the magnitudes of long-range components, the components with penetration included do not form repulsive walls on potential energy surfaces. The main reason for the repulsive behavior is electron exchanges between interacting systems. The exchanges are not due to penetration and in fact extend to all separations *R* between monomers, although they decay exponentially. The charge-penetration (charge-overlap) effects also decay exponentially, so the relative importance of both effects changes in a similar way with *R*, but the physical mechanisms of the two interactions are completely different.

**Answer:** It was never our intention to downplay the general importance of exchange repulsion for intermolecular interactions. We agree that the two interaction terms both are important, and that they have different physical origins. The abstract has been rephrased to clarify that the statement regarding the higher importance of charge penetration compared to exchange for the repulsive character of the interaction energy refers specifically to the investigated Br^–^···HF complex. However, it is interesting to note that the exchange repulsion generally appears weak for the interactions between hydrogen bond donors and Br^–^. This is indicated by our computed potential energy curves, which, for the exception of Br^–^···HF, show that the total interaction energy ($\Delta E_{Int}$) lacks a repulsive character even at short distances; the reason for the minimum in the complexation energy at the equilibrium distance is entirely due to the increasing nuclear deformation energy at short distances. Moreover, this observation is not pertinent to the PC model as these energies are computed fully quantum chemically using the M06-2X method without invoking the PC approximation. It would be highly interesting to investigate the reason behind the weak exchange repulsion in these systems by means of SAPT or supermolecular EDA.

7. **Szalewicz asks:** *Deformation energy:* The authors consider both (vertical) interaction energies and the “complexation energies” that differ from the former by being computed relative to monomers in their gas-phase equilibrium configurations (rather than the same configurations as in the dimer). It has been pointed some time ago [41] that the latter energies are not a useful concept in theory of intermolecular interactions and the paper would be streamlined if these quantities are omitted. For flexible monomers, the use of the latter definition prevents separation of the total system energy into parts due to intermonomer interactions and those due to intramonomer interactions. Also, in symmetry-adapted perturbation theory (SAPT), only the former definition can be used. One sometimes argues that the latter definition is needed to compare with experiment, but in fact interaction energy cannot be measured in any direct way. The experimental complexation energies are measured between ground-state rovibrational levels of the dimer and monomers.

**Answer:** We agree that for weak intermolecular interactions it may be appropriate to neglect the deformation energy and base the analysis on the interaction energy. However, in the type of complexes that we analyze, the deformation energy is large at intermolecular distances close to the equilibrium distance and plays a significant role in determining the shape of the potential energy surface. As already discussed, in many of these systems there is no minimum in the interaction energy ($\Delta E_{Int}$) if the nuclear deformation energy ($\Delta E_{Nuc}$) is neglected. The high strength of the interactions also means that the complexes are well-described by a harmonic force field and that the energies of the accessible rovibrational states are readily obtained by the addition of rovibrational corrections to the complexation energy.

**Szalewicz asks:** Dispersion energy: The authors define dispersion energy as: “Dispersion, or London interaction, is often described as a Coulombic interaction arising from the instantaneous and mutual polarization of the charge distributions of the interacting molecules, e.g. induced dipole–induced dipole interaction.” While this definition is indeed often encountered in the literature, it is not, in my opinion, characterizing correctly the dispersion interaction. In particular, the interactions between induced dipoles from different monomers are a part of the third-order induction energy. I refer to my contribution for another definition of the dispersion energy, which in my opinion better describes the physical character of the dispersion interaction.

**Answer:** We thank Szalewicz for this comment, and we like to emphasize that there are indeed alternative descriptions/interpretations of dispersion. The interested reader is encouraged to read Szalewicz contribution but also other recent articles on this topic, e.g. ref. [42].

**Szalewicz asks:** Dispersive force: The authors write: “as demonstrated by Feynman, the dispersive force can be calculated from the electron density of the molecular complex and is the result of a polarization of each interacting fragment”. What Feynman showed is that forces on nuclei of any molecular system can be calculated from Coulomb force law involving the total electron density of the system ρ(r). However, in my opinion, Feynman’s theorem does not imply that dispersion interaction is Coulomb interactions since the dispersion contribution to ρ(r) is not due just to Coulomb’s law.

**Answer**: The intention was neverto imply that dispersion is a Coulomb interaction in the classical sense. However, in our opinion it is relevant to point out that the Hellmann–Feynman theorem is valid also for dispersion interactions and implies a polarization (deformation) of the densities of the interacting fragments. This density deformation is not in agreement with the description of dispersion as the result of the interaction between instantaneous dipoles (see the previous comment by Szalewicz), as that description suggests that there is no static change in the electron density. In this revised version of the manuscript, we have rephrased the statement to make it clear that dispersion is not a classical polarization interaction.

**Popelier asks:** How universal would be a successful scaling factor between the complexation energy and the point-charge-model interaction energy, beyond the hydrogen-bonded and halogen-bonded systems investigated so far, and including other classes of van der Waals complexes?

**Answer**: A preliminary study has indicated that the scaling factor is surprisingly universal [40]. We have investigated a data set consisting of 138 halogen- (38) and hydrogen- (100) bonded complexes. The X-bonded complexes had donors of the type C-Br and the acceptors were Br^-^ and NH_3_, whereas the H-bonded complexes had donors of the types CH, OH and SH, and the acceptors were Br^-^, Cl^-^, NH_3_ and HCN. The partial charges of the neutral acceptors were obtained by fitting to the electrostatic potential. These whole set data are very well described by a single scaling factor of 0.85. The result indicates that a scaling factor of 0.85 works well for intermolecular interactions between molecules of high or medium polarity. However, the strength of interactions that involve molecules of very low polarity and therefore likely to be dominated by dispersion is expected to be underestimated by such a relationship.

**Popelier asks:** Over which distance range was the (assumed) least-squares fit carried out to obtain the scaling factor? Does the range cover R_o_ to 5 Å, or which fraction of R_o_ if not unity?

**Answer:** The least square fit was carried out for the equilibrium distance R_o_. As can be seen from the full energy curves (Fig. 3 and 4), the scaling factor is always close to unity at distances above 3.5 Å. As discussed in the article, below 2.5 Å the scaling factor gradually decreases (although better phrased as an increasing deviation of the interaction energy from the PC interaction energy) with decreasing distance because of the increasing contributions from Pauli repulsion and charge penetration.

**Popelier asks:** A point charge of -1 to represent the Br^-^in the systems investigated does not differentiate this anion from any other singly charged one, even if their polarisabilities differ a lot. How can such an integer point charge be sufficient to cover the complexity of complexation energy, which should contain mutual polarisation of the “monomers”?

**Answer:** First of all, it is important to remember that we calculate the PC interaction energy ($\Delta E^{PC}_{Int}$) at the equilibrium geometry of the complex obtained with the full quantum chemical model. Thus, the interatomic distance is shorter for smaller ions and consequently the interaction energy will be lower in the PC model due to the distance dependence of Coulombs law. The polarizability of the ion on the other hand increases with size and thus to some extent counteracts the lowering of the interaction energy for the smaller ions. The result appears to be that scaling factor is relatively independent of the ion, although we see a small decrease in the optimum scaling factor going from Br^-^ to to Cl^-^, e.g. from 0.85 to 0.82.

**Popelier asks:** What is the deeper reason for the existence of a scaling factor between the complexation energy and the point-charge-model interaction energy?

**Answer:** We have not made a thorough analysis of the reason for the scaling factor and the answer is therefore somewhat speculative. It can be easily seen that from the energy curves (Fig. 3 and 4) that the individual energy components, including the full interaction energy, have similar functional forms for all systems, and to change the energy curves from one system to resemble another system is just a matter of scaling the individual components. This is expected from a theoretical standpoint as the individual energy components are well defined physically and are described by the same expressions in all systems, see, e.g. Szalewicz’s paper for the corresponding expressions in SAPT. At the energy minimum, the attractive forces are counteracted by the repulsive forces leading to a zero total force. Considering that the functional forms of the attractive and the repulsive energy components always are similar, it can be anticipated from mathematical considerations that the interaction energy at the energy minimum can be approximately expressed by a universal scaling of the attractive energy components. This is easily recognized by analyzing a simple energy expression, e.g. the Lennard-Jones potential where the scaling factor between the attractive energy and the total interaction energy at the minimum is 0.5. Applying this type of reasoning to our systems, it is not surprising that total interaction energy is well described by a scaling of the dominating attractive energy terms, i.e. electrostatics and polarization, considering that the remaining energy terms, in particular Pauli repulsion and dispersion, always follow similar distance-dependent energy expressions.

**Popelier asks:** Can one explain in simple (chemical) terms why a [XHX’]^–1^ system becomes largely symmetrically “hydrogen bonded” with a large degree of covalency only when X and X’ are the same halogen?

**Answer:** We do not have a simple explanation for this behavior, besides the obvious answer that only the [XHX]^–1^ type of system has a strictly symmetrical potential. There are some recent studies analyzing the [FHF]^–1^ system both by experimental and theoretical means that can be consulted but also they lack a simple explanation [17, 43]. When it comes to varying degrees of covalency we discuss that in our answer to the related question of Mo et al. (vid infra).

**Mo et al asks:** **(a)** Do the authors really believe that the (BrHBr)^-^ anion is bound only/mostly by electrostatics? If so, why (HHH)^-^ is a transition state, while (BrHBr)^-^ and (FFF)^-^, etc. are stable species in minima? There are many such examples, which show that creating the impression that electrostatics is a panacea is not the right approach to convince readers that this is an important effect, which exists alongside other effects, all the way to covalency. (BrHBr)^-^ has significant covalency; why not say so clearly?

**Answer:** We do not believe that the (BrHBr)^-^ anion is bound only/mostly by electrostatics, and we have never claimed that it is. What we have tried to do is to investigate how well the interaction energy can be reproduced by only considering electrostatics and polarization. For this purpose, we have used a point charge (PC) model that is completely free from charge transfer as the negative point charge has no electrons that can be transferred. The PC interaction energy ($\Delta E^{PC}_{Int}$) can be rigorously separated into an electrostatic energy and a polarization energy. For the (BrHBr)^-^ anion, the $\Delta E^{PC}_{Int}$ amounts to –39.6 kcal mol^–1^ with electrostatics contributing with –25.3 kcal mol^–1^ and polarization by –14.2 kcal mol^–1^. The total interaction energy ($\Delta E_{Int}$) is very similar to $\Delta E^{PC}_{Int}$, but it should be remembered that the PC model overestimates electrostatics, and the polarization of the HBr unit, due to the neglect of charge penetration. After consideration of charge penetration, we can estimate that the electrostatic interaction energy amounts to 50% or less of the total interaction energy. We agree with Mo et al. that the bonding in not only (BrHBr)^-^, but also to smaller extent in Br^-^···HCl, has a significant covalent character. However, as we are emphasizing in the revised manuscript, a significant covalent character is not necessarily equal to a large charge transfer component. In fact, we believe that there is some degree of covalency, although in most cases relatively small, in the bonding of all the investigated complexes.

**(b)** While $\Delta E^{PC}_{int}$ accurately reproduces both the electrostatic attraction and polarization effect of the HB donors, the repulsive exchange interactions (Pauli repulsion) and the “controversial” charge transfer energy terms are missing in the analysis of the paper. The excellent correlation between $\Delta E_{int}$ and $\Delta E^{PC}_{int}$ is irrelevant to the presence or absence of charge transfer contribution. Back in 2002, one of us found that all energy terms are correlated, and both the polarization and charge transfer energy components can be approximately factored into the electrostatic energy term [44]. This is also reflected in the present paper (Table 1) where the authors recognized the fair correlation (*Rv*^*2*^=0.89) between the electrostatic interaction energy and$\Delta E_{int}$.

**(c)** Within the VB theory, the polarization and charge transfer (CT) in the complex (Br-H-Br)^-^ can be well defined and computed. Have the authors checked the internal correlation of the electrostatic effect with other effects (Pauli repulsion, charge-transfer, i.e. covalency, etc.) using any EDA method?

**Answer:** We agree that the different energy components are likely to correlate, and as we emphasized in our answer to Popelier about the deeper reason for a universal scaling factor, a correlation between the attractive (negative) and repulsive (positive) energy components is expected at the energy minimum. However, it surprised us when investigating both halogen bonded and hydrogen bonded systems involving anions that there was such a poor correlation between the electrostatic interaction energy and the polarization interaction energy. Furthermore, in both type of systems the correlation with the full quantum chemical interaction energy improved dramatically and went from good to excellent when polarization was added to the electrostatic energy. This is no proof *per say* that charge transfer is unimportant but it indicates that if the charge transfer contribution is significant it is likely to correlate strongly with the polarization energy.

**(d)** What about the electrostatic term derived for all the species from EDA? How do these electrostatic terms compare with the values obtained from the PC model?

**Answer:** In the PC model, we represent the Br^-^ by a negative point charge of unitary magnitude. At distances beyond 3 Å, the point charge reproduces the electrostatic potential of Br^-^ nearly exactly as can be seen from Fig. 1. Thus, at such distances the PC electrostatic energy ($\Delta E^{PC}_{ES}$) will closely reproduce the exact electrostatic interaction energy. It can also be seen from Fig. 3 and 4 that at distances beyond 4 Å, where the polarization energy ($\Delta E^{PC}_{Pol}$) is almost negligible, $\Delta E^{PC}_{ES}$ is very close to the full quantum chemical interaction energy ($\Delta E_{Int}$). Between the 3 Å and 4 Å, $\Delta E^{PC}_{ES}$ + $\Delta E^{PC}_{Pol}$ is very close to $\Delta E_{Int}$.

**(e)** Neglecting CT leads to a simple paradox. One can look at the S_N_2 reaction Y^-^ + CH_3_-X ➔ Y-CH_3_ + X^-^. It is clear here that there is CT from one side to the other. These CT effects are also important in the transition state, and if we consider that effects are continuous and not abrupt, then some CT exists also at the clusters (which are “tetrel bonds”). Similar arguments apply to the process, Br^-^ + H-Br ➔ (BrHBr)^-^ ➔ Br-H + Br^-^, or to the F_3_^-^ example (above). Denying the presence of CT is not scientifically sound.

**Answer:** We do not want deny the presence of CT, but rather emphasize that our studies have shown for some particular cases where a large charge transfer component could have been expected that the total interaction energy is well recovered by only considering electrostatics and polarization. The intention has not been to disprove CT, but rather to test how well a model that neglects charge transfer can reproduce the full interaction energy. In fact, our original objective for using the PC model was to demonstrate the need for a separate CT term. However, the results proved our hypothesis to be wrong.

**(f)** Ignoring the Pauli repulsion is not scientifically sound. It is the key driver for the onset of most of the other effects which come out of EDA or VB/BLW analyses.

**Answer:** We do not deny the presence of Pauli repulsion either. However, the full $\Delta E_{Int}$ curves show that in these systems Pauli repulsion must be relatively weak since for all systems, except HF, the $\Delta E_{Int}$ curve never becomes repulsive, not even at very short Br-H distances. Furthermore, for the HF complex the repulsive character of $\Delta E_{Int}$ at short distances can be attributed mainly to charge penetration as shown in Fig. 5.

**(g)** Our final question is, while the PC model works well for charge systems where the electrostatics rules, how and whether can the conclusion derived these systems be applied to neutral systems where noncovalent interactions are ubiquitous?

**Answer:** We originally looked at charged systems because we anticipated that the charge transfer would be particularly large in them. As discussed in the answer to the first question of Popelier, we have found that the PC model works equally well, or better, for systems where the hydrogen bond acceptor is a neutral molecule. However, in such systems we put an ESP-fitted point charge on each atom of the hydrogen bond acceptor. In addition, in the neutral system the polarization contribution is much smaller and there are excellent correlations between $\Delta E^{PC}_{ES}$ and $\Delta E_{Int}$.

(**h)** One of us found pages 15 and 17 to be unclear (e.g. on p. 15–16: “the question remains whether a model that allows for full polarization of both Br^-^ and HBr in the geometry of the (Br-H-Br)- complex would reproduce the charge distribution of the (Br-H-Br)^-^ complex as well as its interaction energy. However, it is clear that such a model would no longer allow polarization to be distinguished from charge transfer.”). Such a statement is confusing and is basically incorrect since VB and BLW do distinguish between polarization and CT. (Similar confusion in the statements exist on page 17 – which tries to force the negation that CT exist and is not just polarization).

**Answer:** We agree that the statement taken out of its context may seem confusing, and in the revised manuscript the passage has been revised as follows: Still, the question remains whether a model that allows for full polarization of both Br^-^ and H-Br in the geometry of the (Br-H-Br)^-^ complex would reproduce the charge distribution of the (Br-H-Br)^-^ complex as well as its interaction energy. However, such a model would require an extremely large and diffuse basis set for each fragment, which would make it impossible to distinguish polarization from charge transfer.

When it comes to distinguishing charge transfer in the VB and BLW methods, Mo et al. themselves emphasize in their article that these methods are dependent on atomic basis functions and they state: “In the extreme scenario, infinite one-centered Gaussian functions can be used for a molecule, such that the basis set limit is reached. Obviously, with the melting down of the individuality of atoms with infinite basis functions, it would be difficult to differentiate the polarization and charge transfer interactions which are often combined to the term ‘orbital interaction’.”
